# Could global norms enable definition of sustainable farming systems in a transformative international trade system?

**DOI:** 10.1007/s43621-023-00130-0

**Published:** 2023-03-27

**Authors:** Markus Giger, Irene Musselli

**Affiliations:** Centre for Development and Environment (CDE), University of Bern, Bern, Switzerland

## Abstract

This paper aims to support differentiation between sustainable and unsustainable agricultural production, with a view to enabling a transformative agricultural trade system by incentivizing sustainable agricultural production. We argue that transformative governance of corresponding global trade flows will need to provide support to the weaker participants in production systems, above all small-scale farmers in the global South, in order to support their food security and a path out of poverty as well as global environmental goals. The present article seeks to provide an overview of internationally agreed norms that can serve as basis for differentiation between sustainable and unsustainable agricultural systems. Such common objectives and benchmarks could then be used in multilateral and binational trade agreements. We propose a list of objectives, criteria, and benchmarks that could contribute to formulation of new trade agreements that strengthen producers who are currently marginalized in international trade flows. While acknowledging that sustainability cannot be easily measured and defined for all site-specific conditions, we posit that it is nevertheless possible to identify such common objectives and benchmarks, based on internationally agreed norms.

## Introduction and objectives

International trade in agricultural products has increased significantly in recent decades. It is associated with far-reaching land use changes [1] and a wide range of economic, social, and environmental impacts [2]. While one common view is that trade in agricultural products can, in principle, reduce pressure on land resources by moving the production to more efficient production regions [3], analyses also show that goods are increasingly sourced from tropical areas. The result is that human pressure on land resources is rising overall [4]. A recent analysis of the impacts of trade on nine environmentally related SDG targets showed that trade improved the scoring of these SDGs for developed countries, but was reduced for developing countries [5]. Even when there is no consensus on the ultimate balance of these impacts of trade, it is widely held that negative impacts—such as violation of human rights standards, substandard and exploitative labour conditions, deforestation, and biodiversity loss—must be urgently addressed [6]. This is even more important if we consider the far-reaching positive changes needed to enable a transition to a global food system that helps achieve the SDGs.

Many private standards have already been developed to address these issues, and various national and regional policy changes have been proposed and are under discussion [7–9]. Use of sustainability criteria and metrics has also grown considerably in trade regulation. Inclusion of social and environmental provisions in bilateral/regional free trade agreements (FTAs) has become increasingly widespread in recent decades, with varying approaches to trade and sustainable development (TSD) provisions implemented in the EU, Australia, Canada, Chile, Japan, New Zealand, Switzerland, and USA [10]. Some countries and regions have also unilaterally established criteria for imported products and their use. For example, the EU has defined sustainability criteria for biofuels that may be applied towards the EU renewable energy targets [11]. In addition, the EU is considering restrictions on deforestation-related commodities [12].

More recently, “Processes and production methods” (PPMs) have gained renewed interest as a result of the strengthening of trade linkages with labour rights and environmental protection. The new EU trade strategy unveiled by the European Commission explicitly acknowledges the legitimacy of applying PPM requirements to imports, in full respect of WTO rules, based on the need to protect the global environment and/or to address ethical concerns [13]. From this perspective, PPMs can be instrumental in harnessing the positive role of agricultural trade towards achieving the SDG agenda. The well-known arguments against PPMs remain important and must be considered. At least three main arguments have been put forth in this respect [14]. Firstly, PPMs can be exploited to discriminate against competitors. Secondly, PPMs can also force producers to comply with rules and regulations that are not embedded in the local social and cultural context. Thirdly, PPMs are difficult to trace or verify at borders, particularly when they are not identifiable based on distinct characteristics of the traded product.

Against this background, this article aims to review internationally agreed and recognized norms that can be used as a reference to define objectives, criteria, and benchmarks with international legitimacy^1^ Thus, the aim is not to argue in favour or against PPMs, but rather to investigate whether internationally agreed norms exist that can provide a foundation for making distinctions based on the way agricultural products are produced and by whom they are produced. Ultimately, the aim is to support differentiating between sustainable and unsustainable agricultural production, with a view to conceptualizing a food system that is transformative [15] by incentivizing sustainable agricultural production.

Given the current state of the global agricultural system and its impacts, we argue that transformative governance of corresponding global trade flows will need to provide support to the weaker participants in the production systems, above all small-scale farmers in the global south. This in order to support their way out of poverty and food insecurity. At the same time, the global agricultural system should support the production of global public goods, such as climate mitigation, biodiversity conservation, and the protection of global water supplies. The trade governance regime should thus also create disincentives or regulation of activities of large-scale producers that contribute to the destruction of tropical forests and lead to displacement of smallholders and indigenous people.

While acknowledging that sustainability cannot be easily measured and defined for all site-specific conditions and recognizing that the assessment of sustainability is also influenced by stakeholders’ values, we posit that it is possible to identify several common core objectives and benchmarks, based on internationally agreed norms, for use in differentiating the social, economic, and environmental dimensions of sustainability. Such common objectives and benchmarks could then be used in multilateral and binational trade agreements. Accordingly, the present article seeks to provide an overview of internationally agreed norms that are available and appropriate to differentiate between sustainable and unsustainable agricultural systems. Based on this review, we propose a list of objectives, criteria, and benchmarks that could contribute to formulation of new trade agreements that would support producers who are currently marginalized in international trade flows.

The present research began by reviewing the initiatives of international organizations and public, 2,3 as well as the scientific literature, in order to identify elements of agreement and disagreement regarding product differentiation on sustainability grounds. Issues of sustainability standards have been discussed in the scientific literature [16–25]. Private standards have been criticized as being dominated by commercial interests, while still others argue to the contrary [26–29]. Therefore, wherever possible, we have chosen to emphasize internationally agreed objectives and norms, rather than standards developed by private organizations.

Our analysis of the literature generated a list of international norms of relevance, starting with the SDGs, the Principles of Agroecology, human rights norms, and the Voluntary Guidelines on the Responsible Governance of Tenure of Land, Fisheries and Forests in the Context of National Food Security (VGGTs). These were then complemented with important objectives we identified by screening the existing initiatives and voluntary standards, in view of creating a comprehensive and balanced set of objectives, criteria, and benchmarks.

The present article is structured as follows: First, we discuss a number of substantive issues with methods that are relevant to our task, reinforcing our selection of key international norms that should be considered. Secondly, we discuss these norms and explain why they are important. Thirdly, we propose and discuss a resulting list of agreed objectives, benchmarks, and criteria. Finally, conclusions and details from our review are provided.

## Methodological considerations

### A highly diverse range of producers in the global food system

Agriculture encompasses a highly diverse range of goods, produced under very different conditions around the world, using different methods practised by producers. Some of these producers are highly capitalized, large-scale commercial businesses, using tractors, equipment, mechanical harvesters, and large amounts of fertilizers and pesticides. The rest are mainly small-scale—sometimes marginalized—producers, who possess very limited machinery and infrastructure and often use very few inputs other than small hand tools and animal power.

Of the approximately 570 million farms globally, over 80% are small-scale farmers. It has been estimated that, globally, 84% of farms are smaller than 2.0 ha and that they operate on 12% [30] and 24% [31] of all agricultural land. Notably, in low- and lower-middle-income countries, the share of land operated by small farms is even higher, estimated at 30–40% of all land used by farming systems in such countries [30]. Smallholder and family farms also cannot be simply equated, as it has been estimated that 98% of all farms worldwide are “family” farms—many of them very large, especially in countries like the USA or Brazil [32].

Therefore, it is important to note that small-scale does not automatically mean sustainable^4^ Indeed, the smallholder system is not uniform. The biggest smallholder groups (those in China, India, and other regions in Asia) have become highly input dependent, for example, using comparatively high amounts of fertilizers and pesticides (and achieving yields similar to those in Europe). Further, a definition of 2.0 ha farm size would exclude many larger farms in different regions that are also producing according to relatively high sustainability standards, such as many commercial organic farms. Depending on the region, even resource-poor farmers may require large land areas to maintain diversified livestock-cropping systems in relative marginal regions (e.g. in the Sahel). Finally, other production systems such as pastoralism (Central Asia, South Asia, Sahel, and others) or the production of non-timber forest products in forested regions simply do not fit definitions based on farm size.

Indeed, it is not possible to make a meaningful distinction between sustainable and unsustainable farms based on only the criterion of size. As a result, it is necessary to focus on a larger set of criteria that should be promoted on behalf of more sustainable systems, including a just transition towards more sustainability. As a result, we will propose to define common principles covering the main relevant dimensions of sustainable production systems. To achieve this, we investigate whether internationally agreed and widely recognized principles exist that can be used to distinguish between farming systems and products that should be supported in a sustainable trade system.

### Addressing ecological, social, and economic objectives

There is a broad consensus in the academic literature, but also in the policy arena and among practitioners, that sustainability in agricultural production needs to consider environmental, social, and economic objectives [15, 34, 35]. It is widely acknowledged that agriculture is a field where ecological, economic, and social processes are highly interlinked. Thus, attempts to distinguish sustainable agricultural production must address a wide range of objectives.

The scientific discussion on sustainability has not produced one universally agreed definition of what sustainability is and how it can be measured [36, 37]. While the world has agreed on a set of SDG goals, the literature also indicates that to navigate the many trade-offs between these goals—considering different spatial and temporal scales, and the different priorities and needs of stakeholders—implies that sustainable development objectives must be defined in specific contexts [38] and stakeholder perceptions are needed to assess whether a system is sustainable or not [39]. This calls for a set of objectives and indicators that can be further defined in context and can be subject to public deliberation.

Therefore, we conclude that differentiation between sustainable and unsustainable systems must be based on normative considerations agreed upon at the international level, and must address the most important social, economic, and environmental dimensions using clear objectives and indicators. At the same time, the definition of indicators must be flexible enough to enable adaptation to individual contexts. Specific benchmarks should be set with caution, only where clear normative agreement exists. The flexibility should enable setting priorities according to each context, and should also enable a focus on the most important improvements in the system. Moreover, adaptations over time should be possible.

### The need to focus on agreed principles at a relatively high level of aggregation

Our study screened initiatives and guidelines of international organizations that aim at developing principles, criteria, or standards in the context of agricultural production and trade. Objectives and standards developed by public and private actors in general address ecological, social, and economic objectives, albeit with different foci and emphases.

A review of existing standards showed that many different standards have already been developed for different purposes and different actors. The International Trade Centre (ITC), a joint agency of the World Trade Organization and the United Nations, maintains a database of sustainability standards. According to its own communications, it currently encompasses over 230 standards initiatives applicable to more than 80 sectors and 180 countries. In 2021, it listed 168 standards related to agriculture [40]. The crops with the highest level of certification are those that are heavily traded, such as coffee, cocoa, tea, and palm oil [41]. Nonetheless, the sustainability impact remains unclear, as most standards focus either on socio-economic or environmental impacts and fail to address trade-offs between them [42]. Many standards do not establish strict criteria or benchmarks, but rather define systems of quality assurance and indicators to measure progress. The vast number of these standards shows that there is a need to focus not on detailed technical definitions and criteria, but on the *underlying principles*, which need to be further defined according to the local context and for specific products.

Against this background, we primarily investigated the most important efforts by governmental organizations, as such principles have already been discussed and formulated on a sufficiently aggregated and globally acceptable level. We sought to focus on those that have already worked based on common principles and standards for many years, have gained a certain level of credibility and standing, and are relevant and of practical importance to our key questions. To address certain areas where agreed standards are lacking, we instead refer to guidelines developed by some of the most important standard-setting bodies from the private sector or public–private organizations or initiatives.

### Issues of measurements, benchmarks, and trade-offs

Once commonly agreed principles and objectives are identified, the *problem of measurement and appropriate benchmarks* remains. What exactly do we measure when some principles or objectives have been agreed upon? Even when a more detailed list of indicators has been agreed upon, a range of more detailed questions typically arises, as many indicators require additional specifications such as the meticulous definition of spatial and temporal scale, benchmarks, and adaption to the context remain. For instance, how could an indicator such as the prevalence of soil degradation be measured? Which definition of soil degradation should be used? Any type of soil degradation and any degree of severity? Or only certain types and beyond a certain threshold of severity? And how is this threshold defined? Do we measure at the plot level, farm level, or landscape level?

*Trade-offs between different objectives***:** A crucial question is how to rate farming systems that have both positive and negative impacts concerning different objectives. Ultimately, this will typically require making trade-offs between the different objectives. What method should be used to give value to ecological and social impacts and compare them to economic impacts? And how should a corresponding scoring system look? Would a minimal score for each dimension be required, or rather a minimal mean of the scores from all dimensions? And do we accept that certain systems exhibit some negative impacts if they seem small compared to their positive impacts?

*Use of negative lists for inputs***:** Neither the SDGs nor the agroecology elements contain negative lists for certain inputs (for instance pesticides) or breeding technologies (GMOs). This is probably due in part to high-stakes commercial interests, but also to diverging assessments of the scientific consensus. However, based on our intention to promote sustainable agricultural systems, some limits to use of certain technologies and inputs could be made if we invoke the precautionary principle and apply it to certain well-known and highly relevant issues, especially GMOs and pesticides.

*How to incentivize transition towards more sustainable practices*: Another challenge is that of deciding how restrictive the criteria should be. For example, being too restrictive by imposing very strong criteria could exclude farms that should be supported for a *transition* towards more sustainability. The objective might be to motivate farmers that currently use unsustainable practices to evolve towards more sustainable practices. In contrast, being too “soft” might enable farms to qualify that are not likely to meet stricter criteria, enabling them to continue with unsustainable practices while benefiting from preferential treatment.

## Review of internationally agreed objectives, norms, and standards

### SDG objectives and indicators

The SDGs are the most comprehensive attempt by the international community to define a set of commonly agreed objectives and targets for sustainable development. As a result, the SDGs serve as our primary reference to derive agreed principles for distinguishing sustainable and unsustainable food systems. In the following, we review these SDG targets and indicators. We list those that are most relevant and best support a selection of objectives and indicators (Table 1). Of particular relevance are the goals (and certain specific objectives and targets) related to food security, employment, and decent work, as well as climate, land, water, and biodiversity.

**Table 1:** List of SDGs and related targets of particular relevance

SDG	Target and selected indicators
SDG 1: End poverty in all its forms everywhere	SDG Target 1.2* By 2030, reduce at least by half the proportion of men, women, and children of all ages living in poverty in all its dimensions according to national definitions* SDG Indicator 1.4.2* Proportion of total adult population with secure tenure rights to land, with legally recognized documentation and who perceive their rights to land as secure, by sex and by type of tenure*
SDG 2: Zero hunger	SDG Target 2.4^a^ *By 2030, ensure sustainable food production systems and implement resilient agricultural practices that increase productivity and production, that help maintain ecosystems, that strengthen capacity for adaptation to climate change, extreme weather, drought, flooding and other disasters and that progressively improve land and soil quality* (SDG Indicator 2.4.1^b^* Proportion of Agricultural Area Under Productive and Sustainable Agriculture)*
SDG 3 Ensure healthy lives and promote well-being for all at all ages	SDG Target 3.*9 By 2030, substantially reduce the number of deaths and illnesses from hazardous chemicals and air, water and soil pollution and contamination*
SDG 5. Achieve gender equality and empower all women and girls	SDG Indicator 5a.1* (a) Proportion of total agricultural population with ownership or secure rights over agricultural land, by sex; and (b) share of women among owners or rights-bearers of agricultural land, by type of tenure. Also: *SDG Indicator 5.a.2 *Proportion of countries where the legal framework (including customary law) guarantees women ‘s equal rights to land ownership and/or control*
SDG 6 Ensure availability and sustainable management of water and sanitation for all	SDG Target 6.4* By 2030, substantially increase water-use efficiency across all sectors and ensure sustainable withdrawals and supply of freshwater to address water scarcity and substantially reduce the number of people suffering from water scarcity *SDG Target 6.5* By 2030, implement integrated water resources management at all levels, including through transboundary cooperation as appropriate*
SDG 8 Promote inclusive and sustainable economic growth, employment, and decent work for all	Several targets (8.5–8.9) are of particular relevance here. Wage rate (a sub-indicator for SDG 2.4.1) is only one dimension of employment and labour standards. Many more issues such as occupational health, number of jobs, labour rights, child labour (SDG Target 8.7), and others play key roles
SDG 12: Ensure sustainable consumption and production patterns	Target 12.2* By 2030, achieve the sustainable management and efficient use of natural resources *SDG Indicator 12.2.1 *Material footprint, material footprint per capita, and material footprint per* GDP Working towards this indicator could imply reducing the use of material inputs in agricultural production, for instance through the use of fertilizers, pesticides, or plastic and other materials
SDG 13 Take urgent action to combat climate change and its impacts	Although changing diets to reduce meat consumption is not listed in any of the SDGs indicators, doing so has been identified as an important strategy to mitigate against climate change—as feed production massively contributes to deforestation (through soy production), as does methane emission from ruminants [43, 44]. With this knowledge regarding the impact of meat consumption on deforestation, SDG 13 points towards the need to reduce large-scale animal feed production, in particular soy production in Latin America
SDG 14 Conserve and sustainably use the oceans, seas and marine resources for sustainable development	is also important, as these waters can also be impacted by agriculture
SDG 15: Sustainably manage forests, combat desertification, halt and reverse land degradation, halt biodiversity loss	SDG Target 15.5:* Take urgent and significant action to reduce the degradation of natural habitats, halt the loss of biodiversity and, by 2020, protect and prevent the extinction of threatened species*

^a^The FAO^5^ is the custodian on behalf of target 2.4, which is of particular relevance to our own research question. The FAO is the most important UN organization dealing with standards and norms in the field of agricultural production. Overall, this target is very broad in ambition and scope. It addresses various wide-ranging objectives, reflecting the complexity of agricultural production as a socio-ecological system. This can be seen when looking at the sub-indicators as defined by the FAO [45]

^b^Indicator 2.4.1 reflects the multiple dimensions of sustainability (economic, environmental, and social). A set of 11 sub-indicators were defined, organized in themes, each mapped to one of the three dimensions. These 11 indicators already map to a large extent the issues involved. In particular, they show that sustainability must include all of the three dimensions. Nevertheless, these 11 sub-indicators, in our view, are not comprehensive, as other SDGs and their targets, as well as other issues and international commitments, should be considered

### Convention on biodiversity conservation

Other more specific targets related to biodiversity have been defined by the *Convention on Biodiversity Conservation* (CBD), known as the *Aichi targets*. Of particular relevance, among others, is Aichi Target 3^6^ which calls for incentives to be created for the conservation and sustainable use of biodiversity. Other targets must also be considered, for instance Aichi Target 7, which calls for sustainable management of agricultural areas. Aichi Target 13 calls for the maintenance of genetic diversity of cultivated plants and domesticated animals. Agricultural systems can also endanger biodiversity-rich areas (tropical forests, and other high-value ecosystems) through the expansion of exploited land areas. These issues are not addressed adequately in SDG 2.4.1 sub-indicator 8 (“Use of biodiversity-supportive practices”). Stronger criteria may be needed here, such as prevention of destruction of such ecosystems (Aichi Target 5).

### Principles of agroecology

The *concept of agroecology* (AE) is another important basis on which we can build [15, 46–50]. It has found recognition both in the FAO and the Committee on World Food Security (CFS). Based on a series of regional seminars, the FAO [51] published a set of ten elements of agroecology in 2018 (5^7^ These elements or principles are intended to support countries in operationalizing agroecology. The FAO council^8^ reviewed the ten elements of agroecology in 2019 [53], and finally *“approved the revised version of the Ten Elements of Agroecology (CL 163/13 Rev.1) as a living document”* [54]. Importantly, the CFS cites FAO’s Elements of Agroecology *“as an internationally agreed formulation of the main elements that characterize agroecology”* [55]. The CFS, as it embraces an inclusive approach with all stakeholders, carries considerable legitimacy regarding the governance of the global food system.

The elements of agroecology consider biophysical, social, economic, and cultural aspects in a common and coherent set of basic principles. It is important to acknowledge that these principles provide flexibility to account for context and capacities^9^.The FAO has also elaborated a framework to monitor the transition to agroecology, known as the Tool for Agroecology Performance Evaluation, or TAPE [50, 56]. Mottet et al. [50] provide scales and scores to assess each of the indicators. The concept of AE can be considered as an important reference document to support a set of internationally agreed objectives and benchmarks.

The concept of AE includes many principles that we will not include in our proposed list of objectives and criteria (see chapter 4) mainly because they are difficult to measure, thus complicating establishment of clear criteria and benchmarks.^10^

### Labour, decent employment, and quality of life

As we have noted above, *SDG 8* already provides objectives in this field.

Further, we propose consideration of the following normative documents related to labour rights:

- *UN Declaration on the Rights of Peasants and Other People Working in Rural Areas (UNDROP),* adopted in 2018, affirms the rights of smallholders as an important element, given many smallholders in the world.
- *UN Declaration on the Rights of Indigenous Peoples (UNDRIP)* specifically addresses indigenous people as a group of actors especially affected by many forms of land use for agricultural purposes in the context of trade.
- *ILO Declaration on Fundamental Principles and Rights at Work (1998),* as amended in 2022, commits the Member States to respect and promote the following fundamental labour principles and rights:
  o freedom of association and the effective recognition of the right to collective bargaining;
  o the elimination of all forms of forced or compulsory labour;
  o the effective abolition of child labour;
  o the elimination of discrimination in respect of employment and occupation;
  o occupational safety and health

Of particular interest are also:

o *ILO Convention 182 (Worst Forms of Child Labour Convention)* that requires countries to take immediate, effective, and time-bound measures to eliminate the worst forms of child labour as a matter of urgency.^11^
o *ILO International Labour Organization Convention 138* (on the minimum age for admission to employment) requires countries to: (1) establish a minimum age for entry into work or employment; and (2) establish national policies for the elimination of child labour.
o *ILO Recommendation 146* stresses that national policies and plans should provide for poverty alleviation and the promotion of decent jobs for adults, so that parents do not need to resort to child labour; free and compulsory education and provision of vocational training; extension of social security and systems for birth registration; and appropriate facilities for the protection of children and adolescents who work.

Private standards regarding employment have defined indicators in more detail and tailored them to the specific requirements of the goods produced. It is important to include such norms in our set of principles. Janker and Mann [16] have analysed 87 farm-related sustainability assessment tools to examine how they operationalize the social dimension. Recurring topics identified were human rights, labour conditions, life quality, and societal impacts. They also identified different approaches to defining criteria. Some use international norms such as human rights and the ILO conventions, others assess farmers’ perception of quality of life. Janker and Mann [16] found a lack of definition of social sustainability and a lack of consensus on what it should entail. They identified human rights and labour conditions as the most feasible for global application. However, they also suggest that while human rights can be used as the bottom threshold (the minimum that must be guaranteed) they are not an appropriate objective to be achieved. They note “The fulfilment of needs, well-being or other perceived life satisfaction might be more adequate approaches, but these are more difficult to operationalize within farm sustainability assessments”. For our purpose, we conclude that human rights and the ILO conventions can be used to set a minimum standard, but that local objectives aimed at improving quality of life and societal impact should be added, as well other objectives defined for the given context. Labour and human rights norms are also recognized in the agroecology principles.

### Land tenure

Land tenure need not only be secure, but also equitable as well as upholding the rights of indigenous people, women, and other potentially marginalized groups such as pastoralists, fishermen, and local communities**.** These principles, also recognized by SDG indicator 2.4.1 and the agroecology principles, have been further described and specified in the *Voluntary Guidelines on the Responsible Governance of Tenure of Land, Fisheries and Forests in the Context of National Food Security (VGGTs)* [57]. The VGGTs, although termed voluntary, have been signed by 123 states, and constitute an important set of commonly agreed guiding principles. Some of the requirements in the VGGT are more complete and more stringent than those mentioned in TAPE. For instance, the VGGTs require that “*Responsible investments should do no harm, safeguard against the dispossession of legitimate tenure right holders and environmental damage, and should respect human rights*” [57], aspects not measured in TAPE. The latter simply calls for participation in land governance.

### Animal welfare

Based on universal ethics principles, the consideration of animal welfare should also be included. According to the World Organisation for Animal Health and its *Terrestrial Code *[58], animal welfare refers to “the physical and mental state of an animal in relation to the conditions in which it lives and dies”. Its guiding principles regarding the welfare of terrestrial animals include the “Five Freedoms” [58]. These principles were developed in 1965 and are now widely recognized. The *Five Freedoms include*: freedom from hunger, thirst, and malnutrition; freedom from fear and distress; freedom from physical and thermal discomfort; freedom from pain, injury, or disease; and freedom to express normal patterns of behaviour [58].

Several existing norms—at the EU level, at the national level, and in certain organic standards (organic, Demeter)—lend further legitimacy to such principles. We identified the European Convention for the Protection of Animals kept for Farming Purposes, as well as the Demeter Standard—well known as having a well-developed and strong standard on animal welfare. We focused on the integration of livestock keeping in farming systems, without opposition to livestock keeping per se. In Switzerland, two well-known standards, incentivized through subsidies, are *BTS* (*particularly high animal welfare housing system*) and *RAUS* (*provisions on regular outdoor exercise*), which both aim to provide animals with more space and the ability to express normal patterns of behaviour. Animal welfare is not captured in the 11 sub-indicators of SDG 2.4.1, nor in the elements of agroecology. (However, TAPE now includes it under social and human values.) Still, livestock keeping and animal welfare can be linked indirectly to agroecology based on its approach to agricultural production and manifold synergies, in particular nutrient recycling [15].

It is generally accepted that animal protein is a key part of the human diet for much of the global population, firmly linking it to SDG 2. Based on this emphasis on synergies as well as nutrient recycling in the agroecology principles, some elements of animal welfare can be addressed, for example based on the need to set limits on livestock numbers and intensity. While we acknowledge the benefits of vegan diets, we believe that based on the current food preferences of most consumers, a modest level of animal protein will foreseeably remain part of a sustainable diet in most regions and for most households.

### Organic agriculture

Under the label of organic agriculture, a lot of important experience, methods, and principles have been developed that are widely known and recognized. Despite its recognition, still only a tiny fraction of global producers practises organic agriculture (2019: 3.1 million producers on 73 million hectares, just 1.5% of global farmland) [59]. At the same time, well-defined and established standards exist, which have officially been recognized or adapted and used in 108 countries, including the US, the EU, Switzerland, and many developing countries [59]. At the international level, the Codex Alimentarius Commission has published guidelines for the Production, Processing, Labelling, and Marketing of Organically Produced Foods [60] and IFOAM has published norms for its Organic Guarantee System [61]. These guidelines and norms offer principles that have proven widely applicable and useful. According to IFOAM, organic agriculture is based on four principles: the principle of health; the principle of ecology; the principle of fairness; and the principle of care. Accordingly, many of the principles of organic production are similar to those of agroecology.

There is, however, no agreement among major stakeholders as to whether the organic system is the only or the most appropriate way to define sustainable food systems. These standards cannot be used as a simple template to formulate criteria, as they apply to a specific model of agriculture that is not necessarily acceptable and adaptable to every context [62]. Organic standards and the corresponding system of certification have been criticized as too costly for small producers. Others have criticized it as being too rigid and not giving enough room for innovation. However, the cost of certification can be reduced through group certification and Participatory Guarantee Systems (PGS) with locally focused quality assurance systems. Indeed, PGS are growing in importance, and are appropriate for small farmers [59, 63–66].

In our view, the definition of a sustainable food system should include organic farming, but be broader and further encompass additional criteria, which we derive from internationally agreed norms.

### Elements of disagreement in setting international norms

#### Genetically modified organisms (GMO)

*Genetically Modified Organisms (GMOs*) are typically used in large-scale monocultures, with the important exception of GMO cotton, which is also used by smaller farmers^12^ The benefits and risks of GMO crops are still contested in varying degrees by consumers and farmers in the countries they are used, with several countries even instituting bans (19 of 27 European Countries have partial or full bans [67]).

Debates on the impacts and risks of GMOs remain ongoing. For instance, the Committee on World Food Security (CFS) report on Agroecology [68] regarding GMOs states***:**** “The World Health Organization (WHO) confirmed that existing regulations have ensured that GM foods currently on the market entail no confirmed health hazards but cautioned against overextrapolation”.* The same report also says*: “In other words, these major health authorities all confirmed the need for further safety testing and evaluation of GM foods on a case-by-case basis. Other scientific assessments have noted the lack of scientific consensus on GM safety, and have called for ongoing, rigorous, and unbiased testing of biotechnology food and food products *[69, 70]*.”*

As long as there is no consensus on the safety risks of GMOs, it can be argued that such products and the farming systems based on them should not be included in preferential trade agreements. The precautionary principle can be used to support this argument. At the same time, others may say it is ultimately a political decision of how to weigh the risks and possible benefits of GMOs. However, the prospects of this technology remain contested among specialists, farmers, and consumers. As a result, differentiating between sustainability and non-sustainability in this area could only be based on current regulations in concerned countries, probably using more restrictive regulations as the benchmark.

#### Pesticides

Issues arise regarding pesticides and other harmful substances used in agriculture. There is no scientific or societal consensus regarding the risks of pesticides in general. Several international assessments on agriculture have not been able to provide clear indications on the best policies regarding the use of pesticides—the views of different stakeholders diverge widely on these issues [34]. While there is no universally agreed list of harmful and potentially harmful substances, inventories exist which could be used as a basis for determination of actual or perceived harms.

Several lists of the most harmful substances exist, and those substances deemed to be most dangerous have been banned by local and national regulations, and also via international conventions. However, the lists established by internationally agreed conventions or agreements have often been criticized as lacking in comprehensiveness. Therefore, certain organizations have created more comprehensive lists of harmful and potentially harmful substances. In particular, PAN International (Pesticides Action Network) [71]^13^ an international NGO, currently maintains the most comprehensive list^14^ of 534 hazardous pesticide active ingredients or groups of active ingredients. It shows that countries differ greatly with regard to pesticide bans: the EU and UK have banned 195 pesticides, whereas others have banned very few—for example, only 14 pesticide substances are banned in Kenya. According to the information we reviewed, neither the FAO^15^ nor the Rotterdam Convention has published a similarly detailed list of HHPs or substances^16^ The WHO has a classification of pesticides by hazard [72]^17^ which could also be used. A Red List can be downloaded from PlantWise (CABI) which lists “Class Ia and Ib Pesticides” according to the WHO Recommended Classification of Pesticides by Hazard. It also includes pesticides that have been banned or restricted by the Rotterdam Convention on the Prior Informed Consent Procedure for Certain Hazardous Chemicals and Pesticides in International Trade, the Stockholm Convention on Persistent Organic Pollutants, and the Montreal Protocol on Substances that Deplete the Ozone Layer [73]. A long list of hazardous materials has also been developed by Fairtrade. The Fairtrade list includes materials that are identified as “highly hazardous” by the Code of Conduct on Pesticide Management adopted by the FAO and WHO in 2013. The list also includes information from the PAN International List of Highly Hazardous Pesticides (HHP) [74]. In the absence of internationally agreed guidelines, we recommend using the Fairtrade list of pesticides that should not be allowed in the agricultural systems promoted.

It is also important to note that very strict rules could also put smallholders at a disadvantage. Firstly, it could be costly for them to implement systems that prove such pesticides are not used in production. Secondly, safer pest control substances may not be affordable to them. Finally, misleadingly packaged dangerous products can easily be used by mistake when farmers are not trained. However, strong regulations can help to protect farmers from endangering their health, by keeping such products out of the market. To be effective, these regulations should be coupled with measures that support farmers to meet their obligations, and not place the cost of compliance on their side. Such issues need to be considered when proposing appropriate rules for preferential treatment.

## Proposed objectives, criteria, and benchmarks

**Fig. 1:**
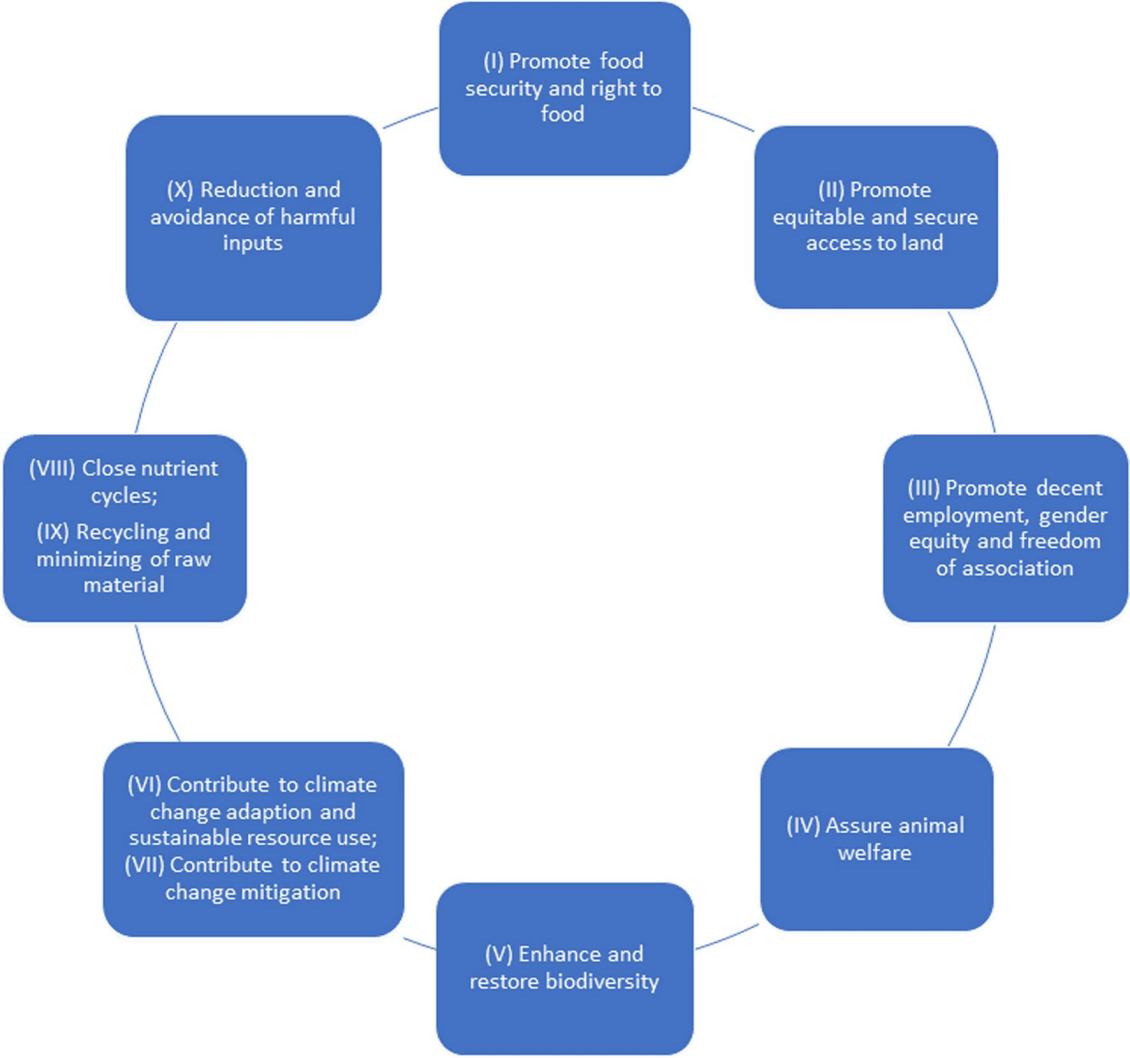
Synoptic view of the objectives proposed. Note that some of the objectives have been grouped in the same text box to reduce complexity of the figure

The result of our investigation is a list of proposed objectives, criteria, and benchmarks *based on internationally agreed objectives and norms* (see Table 2). The objective is to distinguish *sustainable agricultural production systems* for preferential treatment in the framework of a sustainable trade system. In the following section, we discuss these results. We have clustered these objectives around the major objectives of relevant international norms discussed above. A synoptic overview of the result of our investigation is presented in Fig. 1.

(I) **Promote food security and the right to food**
  The Right to Food as a human-rights norm defines specific state obligations to protect it. At the same time, SDG 2 (chapter 3.1) provides a strong justification for inclusion of food security in the criteria of sustainable food systems. When agricultural goods are produced for international markets, there can be a trade-off in terms of land used which could otherwise be exploited for smallholder self-consumption or production of food for the domestic market. Furthermore, there may also be ambiguous effects on land rights and gender equity. In many contexts, however, locals need higher agricultural incomes in order to complement self-produced food, as well as covering cash flow needs for schooling, healthcare, and additional necessities. Notably, export crops often fetch relatively high prices compared to domestic sales, and many smallholders react accordingly to these price signals.
  This export vs. domestic price dilemma has long been discussed within the fair trade community [75, 76]. Research in West Africa has shown that when export production is aimed at high-value crops, the income benefits can outweigh the loss in potential land for domestic food production [77]. However in a meta-review, this positive relationship between certification, farmers' income, and local food security was found to be weak and highly context-dependent [76]. One problem affecting the potential benefits of fair trade standards are the high cost of certification. Therefore, other types of certification such as PGS may be important tools for consideration.
  We conclude that an objective of increased food security is important to include in our list of criteria. Nevertheless, food security is a complex issue and has different dimensions including availability, access, utilization, and stability [78, 79]. The FAO proposes the Food Insecurity Experience Scale (FIES) as a specific sub-indicator to monitor SDG 2.4.1. This approach is now used by the FAO to monitor Food Insecurity on a regular basis [80, 81]. Monitoring this indicator and others related to food security can contribute to developing concrete benchmarks for each context.
  The agroecology concept also emphasizes culture and food traditions. Agroecology also supports food security and nutrition through its contributions for healthy, diversified, and culturally adapted diets. Such aspects could also influence local definitions of these indicators, but it would be difficult to include them in a binding form in the list of indicators.
(II) **Promote equitable and secure access to land**
  Given highly unequal access to land in most countries and lack of other employment opportunities, smallholders’ rights to keep or obtain access to land is a criterion that must be fulfilled. This objective can be monitored using the VGGTs (chapter 3.5), which detail the issues, list many recommendations, and state obligations related to sustainable and equitable land tenure. Of particular importance are issues of land access on behalf of smallholders, pastoralists, traditional communities, and indigenous communities. Adherence to these principles will in many contexts exclude large-scale monocultures and plantations that restrict or exclude access to land for such land users.
(III) **Promote decent employment, gender equity and freedom of association**
  The 1998 Declaration on Fundamental Principles and Rights at Work of the ILO defines a baseline reference for core labour standards (see above, chapter 3.4) and offers a well-defined compendium of objectives and criteria for preferential treatment in the trade context. While not all states have ratified the corresponding core ILO conventions, virtually all have committed to account for the principles enshrined in the 1998 Declaration.
  Trade can be leveraged in various ways to promote the effective implementation of core labour and social commitments, including through the inclusion of labour and social provisions in FTAs and through voluntary standards.
  There has been a “steady strengthening” of labour provisions in FTAs [10]: one third of the trade agreements in force and notified to the WTO in 2019 contain clauses committing the countries party to the agreements to adhere to national and/or international labour standards [82]; the EU, Australia, Canada, Chile, New Zealand, Switzerland, and the USA routinely include labour and social clauses in their FTAs, in separate labour chapters or TSD chapters [10]. They vary widely in scope and stringency (ibid). Key challenges remain, including issues of implementation and enforcement, and the need to embed internationally agreed minimum norms within more complex considerations of societal impacts and local aspirations (see above, Sect. 3.4). One option to address such constraints is to explicitly link enforcement of labour provisions in FTA with technical assistance programmes designed in partnership with third countries to address identifiable needs in relation to the core ILO, with a view to translate principles into practice.
  Voluntary standards also provide means and tools to foster compliance with labour standards across transnational supply chains. An ILO study [24] has compared five voluntary standards—Fairtrade International, GLOBALG.A.P, Social Accountability International (SAI), Sustainable Agriculture Network (SAN), Rainforest Alliance, and UTZ Certified—and found that they all include some ILO norms but do not comply with their full complexity. In terms of reach, all the voluntary standards tended to focus on large farms and actors in agro-food global supply chains, thus providing little support to small-scale farmers. With the exception of Fairtrade International, the contributions of buyers to the selected schemes were found to be limited to participation in the standard-setting process. Only two (Fairtrade International, UTZ) provided a price premium.
(IV) **Assure animal welfare**
  Ethical considerations demand that animal welfare also be included in the system of objectives (see chapter 3.6). Eating less meat should be an objective for societies that have achieved a high level of food security and currently exceed widely recommended levels of dietary meat consumption [83]. At the same time, animals are also an important source of nutrients in many regions of the world. It is debatable whether a transition to more strict vegetarian diets will be socially acceptable and feasible in the future.
  Many observers view ruminants, in particular, as a very well-adapted way of using the world’s vast areas of rangelands, as has been done by pastoralists for thousands of years. Demeter organic standards, for example, even require certified farmers to keep animals as an integral and necessary part of the farm (chapter 3.6). Animals can also be an important source of manure in some mixed farming production systems.
  We propose inclusion of animal production in the definition of sustainable food systems, but recommend adding specifications such as animal husbandry management, stocking rates, breeding, mutilation prevention, nutrition, and veterinary medicine. Such standards exist but are not the same in each country or for each animal species. One option would be to refer to the best standards in the importing country (i.e. for Switzerland in would be BTS and RAUS, in addition to other standards derived from the Swiss Animal Welfare Act i(2005)–especially regarding breeding, killing and slaughter, the dignity of animals and corresponding transportation. It is also possible to refer to the World Organization for Animal Health and its Terrestrial Code (chapter 3.6)*,* for which guidelines have been developed that could be used.
(V) **Enhance and restore biodiversity**
  The Aichi Targets (chapter 3.2) have defined clear objectives and targets for biodiversity conservation. Unfortunately, the world is not on track to meet these targets. Contributions to achievement of the targets should therefore be an important element of the objectives. It should include on-farm and off-farm biodiversity, and also consider the role of production systems on the landscape level (such as measures to enhance biodiversity conservation through wildlife corridors, riparian zones, and protection or creation of high-value conservation areas).
  Such objectives would need to be agreed upon between producers and governments, and monitored at regular intervals. Products certified under PGS systems that include biodiversity conservation targets (on-farm and off-farm, including wildlife corridors, protection of high-value conservation areas, and others).
  Further support for biodiversity conservation can also be derived from the nature-based solutions (NbS) concept, developed in recent years, and supported by the International Union for Conservation of Nature (IUCN), CBD, and the EU commission. NbS are defined by the IUCN as “actions to protect, sustainably manage, and restore natural or modified ecosystems that address societal challenges effectively and adaptively, simultaneously providing human well-being and *biodiversity benefits”* [84].
  We acknowledge that there is also a land sparing debate. It can be argued that intensive production results in avoidance of deforestation or transformation of other biodiverse areas (e.g. wetlands, savannahs) [85, 86], as less land is needed for the production of the same amount. This debate remains unresolved [87–89]. The net impact on biodiversity is ambiguous, as many species are endemic, and it may depend on where land is put into production, and whether this can be offset by sparing land in other regions [90]. However, we argue that providing better incentives for biodiversity conservation through small-scale producers generates important co-benefits for a range of SDGs, and is therefore highly worthwhile.
(VI) **Contribute to climate change adaption, resilience, and sustainable resource use**
  Climate adaptation and resilience are essential objectives and are of particular relevance, as it is widely recognized that agriculture in the global South is one of the sectors that will be (and already is) affected by climate change. Such objectives should be pursued in any sustainability effort. They need to be tailored to the specific context, and with particular measures, technologies, and approaches defined at the local level. IPPC has identified broad measures, and many concrete applications are already implemented in many different contexts. Such measures should be identified in strategic assessments, piloted, implemented, documented, and monitored. In the context of the UNCCD, SLM technologies and approaches are documented on a global scale. This and other repositories of practice could be used to monitor climate-related objectives.
(VII) **Contribute to climate change mitigation**
  Contributions to climate mitigation such as promotion of carbon storage may be possible. Even more important is avoiding deforestation, destruction of peatlands, and high use of fossil fuels in agricultural production.
  Transport remains an important element of carbon emissions over the total lifecycle of agricultural production [91]. Air freight is energy intensive, but many fresh products nowadays travel with air freight. Should such means of transport be totally excluded from support for sustainable agriculture? Should support be allowed for certain high value products? There is no agreement on this question [92–94]. For certain products, it has been shown that lower energy inputs in production can compensate for higher energy needs for transport to consumers [95], even when they travel long distance by air (e.g. when comparing flowers from Kenya with flowers from greenhouses in the Netherlands). Also, dietary choices (reducing meat consumption) may be more important than reducing distances to markets [96]. We therefore argue that internalizing the external costs of air freight should be achieved by taxing or limiting the carbon emissions of global transportation systems more broadly; at the same time, local-level social and environmental benefits are so valuable that the need for air freight should not rule out intensive systems for sustainable production.
(VIII) **Close nutrient cycles**
  An important issue regarding sustainability in agriculture includes nutrient cycles. In many situations globally, nutrient cycles are not closed, leading to off-site harms and environmental, human, and social costs.
  In some of the world’s most intensive agricultural production areas (e.g. Europe and China), high amounts of fertilizers are used, fuelling eutrophication of waterbodies, contamination of groundwater and drinking water, and emission of greenhouse gases. In other parts of the world, production leads to mining of nutrients in the soil, or soil erosion, and nutrient cycles are not closed. Excessive use of fertilizers without closing of the nutrient cycle leads to high energy consumption for the production of N-fertilizers, or to the unsustainable use of non-renewable sources of mineral fertilizer, also causing environmental harms such as contamination of soils with toxic substances (e.g. uranium, chromium). Clearly, fully closing nutrient cycles will not be possible where products are sent to distant markets, but at least crop residues or manure from livestock keeping can be used in production. Closing nutrient cycles is intrinsically linked to integrating livestock in farming systems, rather than separating these systems, as currently occurs in many agricultural contexts due to excessive specialization (on crop or animal production).
(IX) **Recycling and minimizing of raw material**
  Plastic is widely used in agricultural production and resulting traded goods (e.g. greenhouses, packaging, irrigation equipment, weed suppression). It cannot be easily replaced. The effects of plastic on the ecosystem and on human health are only starting to be fully understood. However, innovation will enable use of more biodegradable materials and further minimize the use of plastics [97, 98].
(X) **Reduction and avoidance of harmful inputs**
  It is an important objective to reduce the risks emanating from exposure to hazardous chemicals and from the pollution of air, water, and soils. Farmers and consumers will benefit from the reduction of such risks. As noted in chapter 3.8 in the section on pesticides, internationally agreed lists exist only for a limited number of the most dangerous substances. Nevertheless, the objective should be to further reduce these risks. Orientation can be gained from different sources such as the practice of organic agriculture (chapter 3.7); the principles of agroecology (chapter 3.3.); various lists from international governmental organizations, NGOs, fair trade associations, and research institutions; and the most advanced regulations by importing and exporting countries. Closing nutrient cycles (objective VII) and sustainable resource use (objective VI) will also contribute to this objective.

**Table 2:** Objectives, criteria, and benchmarks to differentiate sustainable agricultural production systems *Note: AE* = *10 Principles of Agroecology (FAO-CFS)*

Proposed objectives	Internationally agreed objectives	Norms of reference	Criteria and benchmark for inclusion	Criteria and benchmark for exclusion	Comments on criteria	Indicators	Reference/additional sources of information
(I) Promote food security and right to food	Right to food, reduce hunger	SDG Indicator 2.4.1, Right to food, AE	From areas with sufficient land resources, or where competition with food can be compensated via high-income earning potential	Must not compete with food security, but rather support food security synergistically (by creating income and producing food for subsistence)	High-value crops such as coffee, cacao from diversified smallholder production (such as agroforestry), and/or from diversified and ecologically rich areas Capacity in the food system to produce, process, store, and provide access to food is strengthened	FAO: Food Insecurity Experience Scale (FIES) Availability of food at affordable prices Sufficient land for food products remains available	Organic coffee Fairtrade IFOAM USAID organic EU organic Organic products from PGS systems http://www.fao.org/3/a-br441e.pdf
(II) Promote equitable and secure access to land	Access to land for all	SDG 1 Indicator 1.4.2, SDG 2, Indicator 2.4.1. Sub-indicator 11, VGGT, AE	From areas with secure land rights for smallholders, pastoralists, indigenous people, women, or from areas where land rights of marginal users can be supported through inclusion in a preferential system of trade	Must not compete with land rights of smallholders, pastoralists, or indigenous people Must not be produced in illegally logged areas	Excludes in practice soy production from Brazil (as legal deforestation cannot be proven in most cases)	Land rights and laws are respected Land rights provide security for small land users Transparency on large-scale land concessions is provided Countries provide evidence that they comply with VGGTs Rights of indigenous people are respected	https://www.rspo.org Large-scale land acquisitions are transparent and can be monitored through land inventories such as www.landmatrix.org or equivalent national databases
(III) Promote decent employment, gender equity, and freedom of association	Fundamental principles and rights at work: Freedom of association and collective bargaining; Elimination of forced or compulsory labour; Abolition of child labour; Non—discrimination in employment and occupation; Occupational safety and health	SDG 1 (Poverty) UNDROP ILO Declaration on Fundamental Principles and Rights at Work ILO Conventions 138 and 182; ILO Recommendation 146; Universal Declaration of Human Rights, Article 23 (ILO Conv. 95 and 131, ILO Rec. 131 and 135)	National laws and practice comply with fundamental principles and rights at work	Violation of fundamental principles and rights at work and failing to carry out the recommendations of the ILO’s investigative procedure	Exclude gross violations of fundamental rights and principles at work The assessment can be sector-specific	Steps taken in law and practice to implement and enforce fundamental labour rights and principles	ILO review process (reports of the Committee of Experts and of the Conference Committee on the Application of Standards ILO’s investigative procedure (List of Commissions of Inquiry and Complaints under Article 26)
(IV) Assure animal welfare	Animals kept enjoy the “five freedoms”	OIE standards and principles Organic Farming Regulation, European Convention for the Protection of Animals Kept for Farming Purposes	Improve animal welfare delivering on the five freedoms and related OIE standards and principles, including through capacity building programmes, and supporting voluntary actions in the livestock sector to improve animal welfare	All products not corresponding to high standards of animal welfare and from farms not well integrated into closed nutrient cycles are not included	Standards must be adjusted to the context Standards must take into account the circumstances of small producers, who cannot comply with the same measures with sanitary and other measures as large industrial livestock systems	Evidence on livestock management provided by farmers or government Evidence on measures to develop and introduce alternatives	OIE (World Organisation for Animal Health) and its *Terrestrial Code*
(V) Enhance and restore biodiversity	Not only reduction of biodiversity losses, but also active restoration of biodiversity	SDG 15, Aichi Targets 3,7,13, AE	Crop diversification and crop rotation Conservation of agricultural landraces and species Establishment of biodiversity-rich production areas. Preservation and reclamation of zones of particular ecological value (wetland, hedges, riparian zones, etc.)	Must not be associated with large-scale deforestation or the destruction of other valuable biomes Must not be associated with monocultures (at a large spatial and temporal scale), which are leading to the deterioration of soils and biodiversity over time	Benchmarks to be defined in the country of origin with the participation of local stakeholders	Evidence on conservation planning and implementation with regard to the concerned production systems (farm or landscape level)	Aichi Targets
(VI) Contribute to climate change adaption, resilience and sustainable resource use	Actions that increase resilience and improve adaption	SDG 13, Paris Declaration	Crop diversification, climate-smart production techniques, soil and water management	Monocultures of annual crops without crop rotation, without adequate soil cover Crop production depleting or polluting water resources, soil management practices that degrade soil quality	Benchmarks to be defined in the country of origin with the participation of local stakeholders	Evidence on adaptation planning and implementation with regard to the concerned production systems (farm or landscape level)	Examples of best practices can be found in the UNCCD SLM knowledge base https://qcat.wocat.net/en/wocat/ IPCC special report on Climate Change and Land [99] IPCC 6^th^ Report 2021 on extreme events [100]
(VII) Contribute to climate change mitigation	Actions that contribute to reducing greenhouse gas emissions or improving carbon storage	SDG 13, Paris Declaration	Promote carbon sequestration in soil and above ground, reduce other greenhouse gas emissions	Must not be associated with large-scale deforestation, depletion of peatland, or highly intensive use of fossil fuel No need for air freight	Benchmarks to be defined in country of origin with participation of local stakeholders Data on transportation from importers and retailers	Evidence on mitigation, planning and implementation with regard to the concerned production systems (farm or landscape level)	Examples of best practices can be found in the UNCCD SLM knowledge base https://qcat.wocat.net/en/wocat/ IPCC special report on Climate Change and Land [98]
(VIII) Close nutrient cycles	Close nutrient cycles to reduce transport, contamination, and eutrophication of water, integration of livestock, production and field crops	AE; Organic Farming	Close nutrient cycles through recycling, composting, integration of livestock and crop production at the farm level or local level	No livestock production without sufficient land where manure can be used productively (on-farm or in exchange with other farms) No livestock production without sufficient land to produce adequate amounts of fodder (at least 90%) on-farm or in the vicinity in exchange with other farms), with exceptions for resource-poor farms	A stocking rate for different livestock species that allows closed nutrient cycles Maximum size of herds Inorganic fertilizers at a low and moderate level complement nutrient deficiencies where no organic manure is available. Standards must consider the circumstances of small producers	Nutrient balance for farms and landscape Evidence on measures to develop and introduce alternatives	IFOAM, but with higher restrictions regarding the transport of manure and fodder than is presently the case USAID organic PGS systems that include this topic
(IX) Recycling and minimizing of raw material	Reduce waste generation through prevention, reduction, recycling, and reuse	SDG 12, Target 12.5 AE	Recycling of material used (plastic, metals, other materials) including nutrients. Reduce fertilizer and pesticide inputs	Excessive use of plastic and materials that cannot be recycled	In addition to health requirements, sanitary standards must consider the need for minimal use of raw materials	Evidence of management of raw materials aimed at recycling and avoidance Evidence on measures to develop and introduce alternatives	[96, 97]
(X) Reduction and avoidance of harmful inputs	Reduce the number of deaths and illnesses from hazardous chemicals and air, water, and soil pollution and contamination	SDG Target 3.9; SDG 2.41, SDG 6, SDG 15, AE (recycling), Organic farming	Management of pesticides. Pesticides on Red List excluded; and Material on Orange List only used according to precise conditions as outlined in Fairtrade List Preferable only substances on the list of allowed substances in organic agriculture	Fairtrade International Red List of Prohibited Materials Any use of substances in CABI List	Consolidated list from different sources, to be defined and regularly updated	Updated list of excluded and restricted inputs Evidence on management (information, regulation, enforcement) Evidence on measures to develop and introduce alternatives	Fairtrade Hazardous Materials List https://files.fairtrade.net/standards/Hazardous_Materials_List_EN.pdf Red List (CABI) https://www.plantwise.org/wp-content/uploads/sites/4/2019/05/Plantwise-Pesticide-Red-List.pdf

## Conclusions

The present study identifies the main criteria and benchmarks that could enable differentiation between sustainable and unsustainable farming systems. This differentiation provides important elements for creation of a more sustainable global agricultural system. The significant progress that has been made towards the creation of internationally agreed objectives and norms makes it possible to establish a list of agreed objectives and to define criteria and benchmarks for inclusions and exclusion. The selection of these criteria is based on these international norms agreed at the global scale. This includes objectives and targets in the framework of the SDGs (chapter 3.1), human rights norms (chapter 3.4), the UN Convention on Biodiversity Conservation (chapter 3.2), the Voluntary Guidelines on Responsible Governance of Tenure of Land (chapter 3.5), the Terrestrial Code (chapter 3.6 on animal welfare), and other important agreements such as the Principles of Agroecology (chapter 3.3), or established practices (3.7 on organic agriculture) that are relevant to our topics.

The proposal lists a set of 10 objectives covering all three dimensions of sustainability, which is necessary for a balanced assessment. We also propose indicators to measure production systems in line with these criteria. While the indicators are generic, they are based on the understanding that they can be refined in more detail for specific sectors and for specific contexts.

When taking a synoptic view of our proposed objectives, it becomes apparent that farming systems that can fulfil these objectives and related criteria will be clearly distinct from large-scale, monoculture operations or massive animal farms that are far removed from their fodder bases. Farm and farming systems that meet these criteria will make important contributions to food security, income creation, and fair employment conditions as well as to the environmental criteria of biodiversity, climate change adaptation, and mitigation. They will recycle as much as possible, limiting inputs of pesticides, and manage soils sustainably.

While the farms that meet these criteria may be very diverse, and exhibit different characteristics depending on the region and context where they are situated, our criteria will help to draw distinctions and differentiate these farms from less sustainable farms. Supporting such farming systems through preferential trade systems could incentivize a transformation towards more sustainable food systems.

Overall, we aim at a transformation towards sustainability of farming systems [15]. With these objectives and criteria, we seek to create synergistic impacts between individual objectives. Our list of objectives should be analysed with this intention in mind, rather than simply considering each objective or criterion in isolation. Promotion of synergies through the different criteria is important, as it would make it possible to foster a “sustainability package” that could incentivize more sustainable practices than those which currently dominate. Ultimately, the positive discrimination we are aiming at should foster development in this direction.

At the same time, the corresponding indicator system should not be made too rigid, as this would slow down innovation. Innovation is needed, as most of today’s production systems have their share of shortcomings—and improvements are both necessary and possible. Related criteria and indicators should be used not only to differentiate between sustainable and unsustainable systems, but also to measure improvements. Within a system aimed at sustainable trade, such improvements should be tangibly encouraged. This does not rule out supporting systems that do not fully meet the proposed criteria, as long as there is credible evidence that their performance is improving over time.

## Footnotes

1 This paper is a revised and improved version of a working paper entitled “Sustainable Farming Systems: Elements of agreement and disagreement in recent international debates” (Giger M., 2022) produced in the interdisciplinary research project “Sustainable Trade Relations for Diversified Food Systems”, financed by the Swiss National Science Foundation (SNSF). The *objective* of the project is to support sustainable food systems worldwide by framing sustainability-related trade regulation. The focus of the project is on the role and leverage of domestic governments. The main research question of the project is: If a domestic government, for example the Swiss government, seeks to engage in sustainable trade relations to promote sustainable food systems, how can it do this? Two specific sub-question addressed in this project are: *A* What policy space does a WTO member state have to differentiate between ‘like’ products in trade measures on sustainability grounds? How is this policy space delineated by guiding principles of other public international law, including the principle of sovereignty? B Which ‘elements of sustainability’ can guide the distinction between ‘diversified farming systems’ of a high sustainability value and other farming systems?

2 Apart from these normative efforts by international organizations, *individual states or groups of states* have also introduced norms that are of relevance here. These include rules for specific production systems (for instance EU organic, USAID organic, Swiss Organic Farming Ordinance 910.18), rules regarding animal welfare, and many specific rules and regulations that have an impact on production (pesticides, environmental regulations, labour standards, etc.). For our study, such norms are of special interest when they are applied similarly by many states (as is the case for organic production).

3 Many private standards and benchmarks have also been elaborated. Some of them are well-established and well known, nevertheless, they often only apply to certain market segments or products and are not (yet) recognized as universal principles defining sustainability. Well-known examples include fair trade standards, private organic standards (for instance BioSuisse), or specific standards for major agricultural commodities such as coffee, cacao, or palm oil (for instance RSPO). Nevertheless, if selected with our objective in mind, they can be used to give credibility and substance to some of the principles and indicators that will be defined. Further, as the example of organic standards or RSPO shows, some of these standards have evolved to a sort of hybrid status, informed and influenced both by private and public actors.

4 A good starting reference is the CFS report on Agroecology [68]. Some quotes from the report, which give some insights into the issues to be discussed: “Farm size is relative, and context-specific, based on historical, social, economic and ecological conditions: for instance, a farm called ‘small’ in the United States of America can be considered as ‘large’ in many African countries. Family farms, however, both in developed and developing countries, may share common features with regard to innovation, agrobiodiversity, intensification strategies and links to territories [33].” Sourisseau J-M. Farming and the Worlds to Come. Dordrecht Heidelberg New York London: Springer; 2014. 1 p. “Yet, diversification is not an exclusive characteristic of small-size farms, nor are all small farms diversified. This suggests that diversification might be explored across a range of small to large farm sizes through supportive public policies, research and civil society initiatives [31]”.

5 Discussions were held with the FAO (Plant Production and Protection Division) to discuss the current work of the organization, and the literature regarding standards and principles for sustainable agriculture was consulted. It became clear that earlier attempts to define good agricultural practices at the global level had stalled, but currently the work focussing on SDG-related indicators and on agroecology has become an important avenue for the FAO.

6 By 2020, at the latest, incentives, including subsidies, harmful to biodiversity are eliminated, phased out or reformed to minimize or avoid negative impacts; and positive incentives for the conservation and sustainable use of biodiversity are developed and applied, consistent and in harmony with the Convention and other relevant international obligations, taking into account national socio-economic conditions.

7 The ten elements are diversity, co-creation and sharing of knowledge, synergies, efficiency, recycling, resilience, human and social values, culture and food traditions, responsible governance, and circular and solidarity economy [52].

8 Composed of forty-nine member nations elected for three years.

9 The CFS notes: “The challenges food systems face are highly complex, context-specific and often unpredictable. Transformation to sustainable food systems is needed, in a coherent manner, as appropriate, and in accordance with and dependent on national context and capacities. There is no single approach for achieving food security and nutrition and all food systems have the potential to contribute further to sustainable agriculture and food systems that enhance food security and nutrition by following context appropriate transition pathways” (55).

10 The following principles of AE are not covered by our list but could be included as transversal requirements to be integrated in eventual mutual agreements between trading partners. (1) Co-creation and sharing of knowledge (2) Circular and solidarity economy (3) Synergies. The principle of Human and social values can be supported through specific international norms, such as the right to food, decent work, occupational health, access to land, and adaption to climate change, as well as animal welfare and biodiversity conservation as intrinsic human values.

11 In 2020, ILO Convention No. 182 became the first ILO convention to achieve universal ratification

12 According to IAASA, 190.4 million hectares of biotech crops were planted by up to 17 million farmers in 29 countries, of which 92 million hectares were soy, 61 million hectares maize, 26 million hectares cotton and 10 million hectares canola (rapeseed). https://www.isaaa.org/resources/publications/pocketk/16/. Accessed 13 Oct. 2021.

13 PAN International Consolidated List of Banned Pesticides 2021. Explanatory note. https://files.panap.net/resources/Consolidated-List-of-Bans-Explanatory.pdf. It contains information from 162 countries “The countries with the most known bans are those of the EU and the UK (175 banned + 208 specifically ‘not approved’ pesticides which are Highly Hazardous Pesticides (HHPs)1 and/or banned by another country), Switzerland (140), Brazil (131), Egypt (76), Saudi Arabia (73), Indonesia (61), Cambodia (60), India (55), Mauritania (52), Palestine (52), and China (51).” It does not include those banned pesticides regarded as being obsolete according to the 2009 WHO Recommended Classification of Pesticides by Hazard.” As a comparison, Kenya is listed with 7 banned pesticides, and USA with 22.

14 https://pan-international.org/pan-international-consolidated-list-of-banned-pesticides/ (accessed 16/6/2022).

15 FAO lists conventions and treaties responsible for such bans https://www.fao.org/pesticide-registration-toolkit/information-sources/restrictions-and-bans/en/.

16 The Rotterdam Convention has a list of pesticides and chemicals that are subject to rules for handling and exportation and importation. Stockholm Convention on Persistent Organic Pollutants has listed the POPs listed for elimination (it evolved from the “dirty dozen”).

17 https://apps.who.int/iris/bitstream/handle/10665/332193/9789240005662-eng.pdf?sequence=1&isAllowed=y (accessed 28.10.2021).
